# The Risk Factors of Refractory Adult-Onset Still's Disease

**DOI:** 10.1155/ijh/6689086

**Published:** 2025-03-17

**Authors:** Lin Cheng, Hexiang Zong, Dongxu Li, Yaqin Zhang, Long Qian

**Affiliations:** Department of Rheumatology and Immunology, The Second Affiliated Hospital of Anhui Medical University, Hefei, Anhui, China

**Keywords:** neutrophil to lymphocyte ratio, platelet, refractory adult-onset Still's disease, risk factors, system score

## Abstract

**Background:** Adult-onset Still's disease (AOSD) is a rare systemic inflammatory disorder of unknown etiology and pathogenesis. Some patients fail to respond to conventional glucocorticoids and immunosuppressant therapies, a condition known as refractory AOSD. The prognosis for patients with refractory AOSD is typically poor, significantly impacting their quality of life and overall health. This study retrospectively analyzes the predictive factors for refractory AOSD to provide new strategies and insights for clinical diagnosis and treatment.

**Methods:** Overall, 105 AOSD patients hospitalized between January 2008 and October 2024 were selected, 41 of whom were classified as refractory. Multivariate logistic regression analysis was conducted to identify risk factors for refractory AOSD, and receiver operating characteristic (ROC) curves were used to evaluate the predictive power of these indicators.

**Results:** Patients with refractory AOSD were more likely to develop splenomegaly and MAS. Additionally, the neutrophil-to-lymphocyte ratio (NLR), lactate dehydrogenase, serum ferritin (SF) levels, and AOSD system score were higher in refractory cases than in nonrefractory cases, while lymphocyte count and platelet (PLT) count were lower in the refractory AOSD group (*p* < 0.05). Multivariate logistic regression analysis identified PLT, NLR, and AOSD system scores as independent risk factors for predicting refractory AOSD. ROC curve analysis revealed that the area under the curve for PLT, NLR, and AOSD system scores were 0.659, 0.661, and 0.660, respectively. The optimal cutoff values for PLT, NLR, and AOSD system score in predicting refractory AOSD were 314.5 × 10^9^/L, 10.555, and 5.5, respectively, with sensitivities of 80.5%, 53.7%, and 75.6% and specificities of 46.9%, 75.0%, and 50.0%, respectively.

**Conclusion: **PLT < 314.5 × 10^9^/L, NLR > 10.555, or an AOSD system score of > 5.5 before treatment may serve as independent risk factors for predicting refractory AOSD, providing clinicians with an early warning to identify disease progression.

## 1. Introduction

Adult-onset Still's disease (AOSD) is a rare, systemic inflammatory disorder of unknown etiology, characterized by high spiking fever, an evanescent rash, and polyarthritis. Alongside these major symptoms, patients often present with other features indicative of multiple organ involvement, such as sore throat, lymphadenopathy, hepatosplenomegaly, and elevated serum liver enzymes and ferritin levels [1, 2]. Glucocorticoids are the first-line treatment for AOSD, and patients generally respond well to these medications, despite the presence of intense systemic inflammation. However, there is no universally accepted standard for glucocorticoid dosage in the treatment of AOSD. Previous studies have suggested initiating treatment with glucocorticoids at a dose of 0.5–1 mg/kg/day, with higher dosages appearing more effective in controlling the disease [2, 3]. Patients receiving higher prednisone doses (≥ 40 mg or 0.8 mg/kg daily) typically experience faster resolution of AOSD symptoms and fewer relapses compared with those on lower doses [2]. The second-line treatments for AOSD include immunosuppressants such as methotrexate and Cyclosporine A. In clinical practice, some patients fail to respond to conventional corticosteroid and immunosuppressive therapies. These patients are classified as having refractory AOSD, although a standardized definition remains lacking [4, 5].

Refractory AOSD is frequently associated with severe complications, including macrophage activation syndrome (MAS), disseminated intravascular coagulation, and liver failure, and often carries a poor prognosis [6–8]. However, the specific risk factors that predispose patients to refractory AOSD remain unclear.

The neutrophil (NEU)-to-lymphocyte ratio (NLR) determined by the respective counts of NEU and lymphocytes, and platelet (PLT)-to-lymphocyte ratio (PLR), calculated as the ratio of PLT count and lymphocyte count, are widely known indicators of the cellular immune inflammation. Currently, studies on these two indicators in AOSD are relatively limited. In this study, we retrospectively analyzed the clinical features, laboratory findings including NLR and PLR, and imaging data of 105 patients with AOSD (including 41 with refractory AOSD) in an effort to identify potential risk factors for refractory AOSD.

## 2. Materials and Methods

### 2.1. Patients

We conducted a retrospective analysis of AOSD patients from the Second Affiliated Hospital of Anhui Medical University, covering the period from January 2008 to October 2024. All patients met the most sensitive Yamaguchi diagnostic criteria for AOSD established in 1992 [9]. The patients with MAS were diagnosed according to the 2004-HLH criteria [10]. Infectious diseases, malignancies, and other autoimmune diseases were excluded from the study. Infectious patients were excluded through blood or bone marrow (BM) cultures, pathogen detection, and chest and abdominal computed tomography (CT). Imaging examinations, including CT, ultrasound, and ^18^F-FDG positron emission tomography/CT scans, as well as BM and lymph node biopsies, were performed to rule out malignant tumors. Rheumatic diseases were excluded based on blood tests, including antinuclear antibodies (ANA), rheumatoid factor (RF), anticitrullinated peptide antibodies, and antineutrophil cytoplasmic antibodies. Clinical data, such as gender, age, disease duration, fever, skin rash, arthralgia, sore throat, myalgia, hepatosplenic lymphadenopathy, internal organ involvement, comorbidities, treatment, and prognosis, as well as laboratory results, including routine blood tests, serum ferritin (SF) levels, liver function tests, C-reactive protein (CRP), erythrocyte sedimentation rate (ESR), D-dimer, fibrinogen (FBG), RF, and ANA, were recorded in detail [11]. All data were collected at the time of diagnosis with AOSD and prior to treatment with glucocorticoids and immunosuppressants. Comorbidities included in the study were hypertension, diabetes, chronic gastritis, chronic obstructive pulmonary disease, cerebral infarction, and coronary heart disease. The study was approved by the Ethics Committee of the Second Affiliated Hospital of Anhui Medical University, and informed consent was obtained from all participants (YX2023-169).

### 2.2. Definitions

The patients were divided into two groups: the refractory group and the nonrefractory group. Currently, there is no universally accepted definition of refractory AOSD. Based on previous reports [2, 3, 12], patients in our study were classified as having refractory AOSD if they showed no improvement in clinical symptoms or laboratory indicators despite being treated with high-dose glucocorticoids (≥ 1 mg/kg per day) and immunosuppressants such as methotrexate and Cyclosporine A, which included a dose of 1 mg/kg per day of prednisone (or its equivalent) for more than 2 weeks, or if it was difficult to taper off glucocorticoids. The refractory AOSD patients included in the study all had systemic flare rather than merely presenting as chronic polyarthritis.

Disease activity was evaluated using the AOSD system score, which includes fever, typical rash, myalgia, pleurisy, pneumonia, pericarditis, hepatomegaly or abnormal liver function, lymphadenopathy, abdominal pain, leukocytosis (> 15,000/mm^3^), weight loss, and gastrointestinal symptoms [13]. Pleurisy, pleural effusion, or pulmonary parenchymal changes were evaluated using chest radiography or chest CT, while pericardial effusion or pericarditis was assessed using echocardiography.

### 2.3. Statistical Analysis

Normal distribution data are presented as mean ± standard deviation (SD). Measurement data were analyzed using the Student's *t*-test in SPSS 16.0 software, while counting data were compared using the chi-square test or Fisher's exact test. Nonnormally distributed data are presented as the median (interquartile range), denoted as M (P25, P75), and analyzed using the nonparametric Mann–Whitney *U* test. Independent risk factors for refractory AOSD were identified through multivariable logistic regression analyses. The cutoff values for these risk factors were determined using receiver operating characteristic (ROC) curves. A *p* value of < 0.05 was considered statistically significant.

## 3. Results

### 3.1. Clinical Characteristics of AOSD Patients

**Figure 1:**
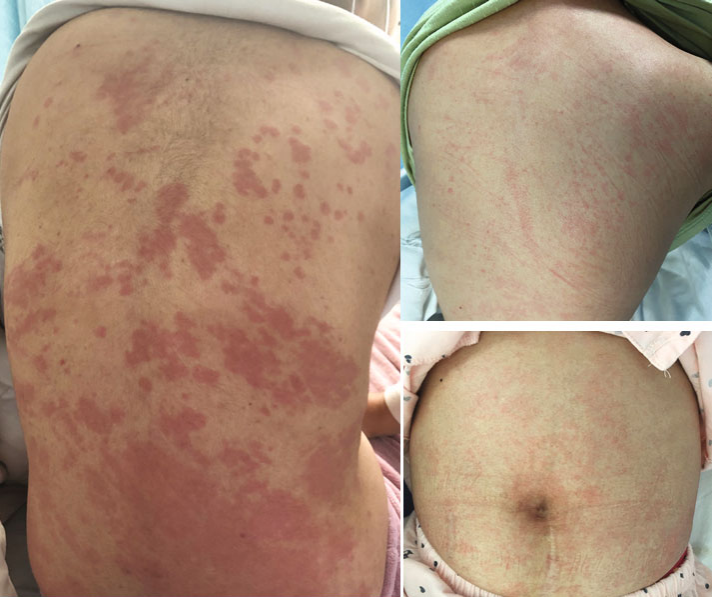
Characteristics of rash in AOSD patients.

A total of 105 AOSD patients were included in the study, comprising 32 males and 73 females. The median age at onset of AOSD was 36 years (range: 27–54.5 years). All patients experienced fever. Among them, 99 (94.29%) had a rash (Figure 1), 80 (76.19%) experienced sore throat and lymphadenopathy, and 59 (56.19%) reported myalgia. Splenomegaly, liver damage, lung and heart involvement, MAS, and comorbidities occurred in 33.33% (*n* = 35), 25.71% (*n* = 27), 40.95% (*n* = 43), 17.14% (*n* = 18), 23.81% (*n* = 25), and 23.81% (*n* = 25) of patients, respectively. Additionally, the following data from AOSD patients were recorded: white blood cell (WBC) count, NEUs, lymphocyte count, NLR, PLT count, PLR, alanine aminotransferase (ALT), aspartate aminotransferase (AST), alkaline phosphatase (ALP), *γ*-glutamyl transferase (GT), lactate dehydrogenase (LDH), CRP, ESR, SF, FBG, D-dimer, and AOSD system score (Table 1).

### 3.2. Clinical and Laboratory Characteristics of Refractory and Nonrefractory AOSD Patients

**Figure 2:**
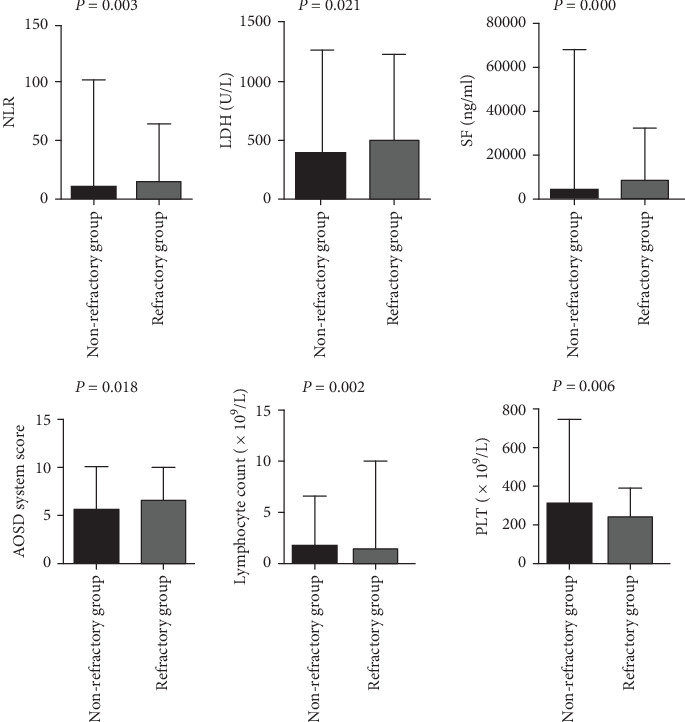
The levels of NLR, LDH, SF, AOSD system score, PLT, and lymphocyte count in the refractory group and non-refractory group. Notes: NLR, neutrophil to lymphocyte ratio; LDH, lactic dehydrogenase; SF, serum ferritin; PLT, platelet.

**Figure 3:**
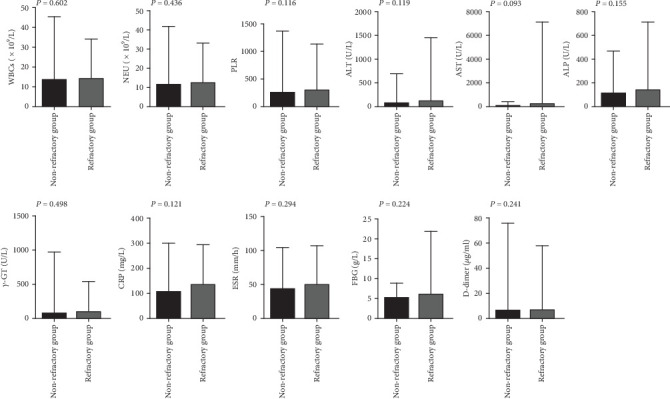
The levels of WBCs, NEU, PLR, AST, ALT, ALP, *γ*-GT, CRP, ESR, FBG, and D-dimer in the refractory group and nonrefractory group. Notes: WBCs, white blood cells; NEU, neutrophil count; PLR, platelet to lymphocyte ratio; ALT, alanine aminotransferase; AST, aspartate aminotransferase; ALP, alkaline phosphatase; *γ*-GT, *γ*-glutamyl transferase, CRP, C-reactive protein; ESR, erythrocyte sedimentation rate; FBG, fibrinogen.

The incidences of splenomegaly and MAS were significantly higher in refractory AOSD patients (48.78%, 60.98%) than in nonrefractory AOSD patients (23.44%,0.00%; Table 2). Levels of NLR, LDH, SF, and the AOSD system score were elevated in refractory AOSD patients, whereas lymphocyte count and PLT count were lower than those in nonrefractory AOSD patients (Figure 2). No significant differences were observed between the two groups in terms of age; disease duration; sex; fever; sore throat; arthralgia; myalgia; rash; lymphadenopathy; lung and heart involvement; comorbidities; and levels of WBCs, NEU, PLR, ALT, AST, ALP, *γ*-GT, CRP, ESR, FBG, and D-dimer (Figure 3).

### 3.3. Multivariate Logistic Regression Predicts Risk Factors for Refractory AOSD

Based on the above results, multivariate logistic regression analysis was conducted on indicators showing statistical significance and clinical relevance, including age, disease duration, comorbidities (present vs. absent), splenomegaly (present vs. absent), lymphocyte count, PLT, NLR, SF, and the AOSD system score. The analysis revealed that PLT, NLR, and the AOSD system score were significant risk factors for predicting refractory AOSD (Table 3).

### 3.4. Predictive Value of Laboratory Indicators in Refractory AOSD

**Figure 4:**
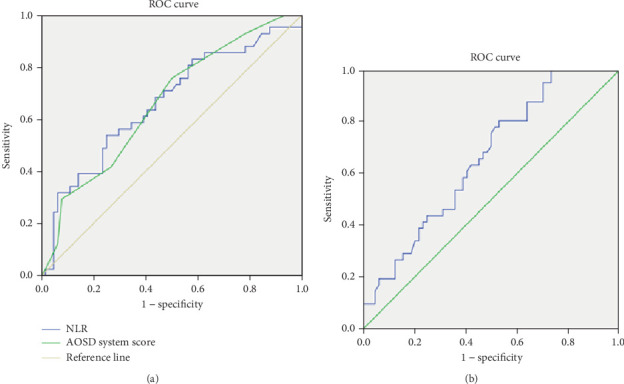
Predictive value of laboratory indicators in refractory AOSD. (a) The ROC curve of NLR and AOSD system score. (b) The ROC curve of PLT.

The optimal cutoff values that best distinguished refractory AOSD from nonrefractory AOSD were determined by maximizing the sum of sensitivity and 1-specificity in the ROC curves. Among the variables analyzed, the PLT, NLR, and AOSD system score, with cutoff values of 314.5 × 10^9^/L, 10.555, and 5.5, respectively, demonstrated the highest sensitivity (80.5%, 53.7%, and 75.6%), specificity (46.9%, 75.0%, and 50.0%), and area under the curve values (0.659, 0.661, and 0.660), respectively, indicating their diagnostic potential for refractory AOSD (Table 4 and Figure 4).

### 3.5. Treatment and Follow-Up of Refractory AOSD

**Figure 5:**
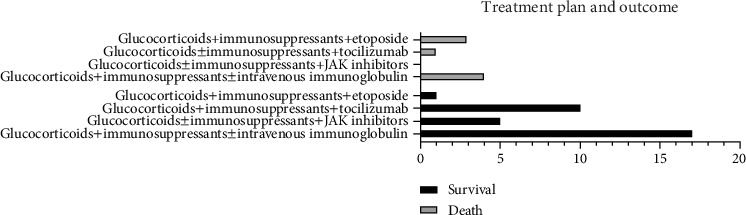
Treatment and follow-up of refractory AOSD.

Of the 41 refractory AOSD patients, 33 survived, and eight died. Among the survivors, treatment regimens included the following: 17 patients (51.52%) received glucocorticoids plus immunosuppressants (methotrexate or cyclosporine) with or without intravenous immunoglobulin; four patients (12.12%) were treated with glucocorticoids, immunosuppressants, and JAK inhibitors; one patient (3.03%) received glucocorticoids and JAK inhibitors; 10 patients (30.3%) were treated with glucocorticoids, immunosuppressants, and tocilizumab with or without intravenous immunoglobulin; and one patient (3.03%) received a combination of glucocorticoids, immunosuppressants, JAK inhibitors, intravenous immunoglobulin, and etoposide. Among the eight patients who died, the treatment regimens included the following: four patients treated with glucocorticoids and cyclosporine with or without intravenous immunoglobulin, three patients treated with glucocorticoids, cyclosporine, and etoposide with or without intravenous immunoglobulin, and one patient treated with glucocorticoids, cyclosporine, and tocilizumab. Six of the deceased patients died from shock, one from multiple organ failure, and one from liver failure (Figure 5).

## 4. Discussion

A total of 105 AOSD patients were included in the study, of whom 41 had refractory AOSD. All clinical factors including splenomegaly, comorbidities, lymphocyte count, PLT, NLR, SF, and the AOSD system score deemed significant in univariate analysis and in clinical were included in a logistic regression model, alongside previously reported [14] potential risk factors, such as age and disease duration. Our analysis revealed that PLT, NLR, and the AOSD system score were independent risk factors for refractory AOSD. Identifying these factors early may help clinicians recognize refractory AOSD and initiate appropriate treatment promptly.

AOSD is a rare inflammatory disease, and currently, there are few international reports investigating risk factors for predicting refractory AOSD. A study by Yin et al. identified age, fever, disease course, PLT, SF, and ESR levels as independent adverse factors for refractory AOSD [14]. Splenomegaly, a common feature in AOSD patients, has been shown to be a risk factor for the development of MAS in these patients [15]. In our study, we found that the incidence of splenomegaly was higher in refractory AOSD patients than in nonrefractory patients. Additionally, the occurrence of MAS was more frequent in the refractory AOSD group, suggesting that refractory AOSD patients may be more susceptible to developing MAS. Further data collection is planned to compare the characteristics of refractory AOSD patients with and without MAS. SF is an acute-phase protein primarily found in tissues such as the liver, spleen, BM, and muscles. Studies have shown that SF levels rise during inflammatory conditions, including AOSD [16]. Hyperferritinemia is a key marker of macrophage activation and is commonly observed in conditions such as AOSD, reactive hemophagocytic lymphohistiocytosis, catastrophic antiphospholipid syndrome, and septic shock [17]. SF levels are crucial for diagnosing AOSD and are considered an important biomarker of the disease. In the present study, we found that SF levels were elevated in patients with refractory AOSD. This may be attributed to secondary MAS in these patients, or it could indicate that macrophage activation is more pronounced in individuals with refractory AOSD.

The NLR is a known indicator of systemic inflammation. Neutrophilia combined with lymphopenia is a characteristic response of the innate immune system to inflammation [18]. Several studies have demonstrated that an elevated NLR can serve as a reliable marker of disease activity in various autoimmune and inflammatory conditions, such as systemic lupus erythematosus, dermatomyositis, and AOSD [19–21]. Seo et al. found that NLR could be used not only as a diagnostic tool but also as a predictor of AOSD recurrence and for differentiating AOSD from HLH [22]. In our study, we observed that NLR levels were significantly higher in patients with refractory AOSD, and multivariate logistic regression analysis revealed that NLR was an independent risk factor for predicting refractory AOSD, with an optimal threshold of 10.555, sensitivity of 53.7%, and specificity of 75.0%. These findings suggest that NLR could be a valuable marker for assessing refractory AOSD. To the best of our knowledge, this is the first report identifying NLR as an independent risk factor for refractory AOSD.

PLT plays a pivotal role not only in hemostasis and thrombosis but also in inflammation [23]. However, there is currently no literature directly linking PLT count to AOSD. Our study revealed that PLT count was lower in patients with refractory AOSD compared to those with nonrefractory AOSD, which may be due to the increased likelihood of secondary MAS in these patients. Additionally, multivariate logistic regression analysis indicated that PLT was an independent risk factor for refractory AOSD, with an optimal threshold of 314.5 × 10^9^/L, sensitivity of 80.5%, and specificity of 46.9%. In a study by Xu et al. [24], adenosine deaminase (ADA) was found to be a biomarker for diagnosing AOSD and assessing disease activity, with ADA levels being inversely correlated with PLT count and positively correlated with levels of SF and AOSD system score. Based on the report [24] and our findings, it is plausible that decreased PLT levels could serve as a potential biomarker for assessing the prognosis of patients with refractory AOSD, especially when the disease is significantly active.

The AOSD system score, as proposed by Pouchot et al. [13], includes fever, rash, pleurisy, pneumonia, pericarditis, hepatomegaly or abnormal liver function tests, lymphadenopathy, splenomegaly, abdominal pain, leukocytosis (> 15,000/mm^3^), sore throat, and myalgia. A previous study indicated that mortality significantly increased when the system score exceeded seven points. Furthermore, it was found that an AOSD score greater than seven points and an SF level exceeding 684 *μ*g/L were predictive of MAS occurrence in AOSD. In our study, we first observed that the AOSD system score was higher in refractory AOSD patients. Multivariate logistic regression analysis further suggested that the AOSD system score is an independent risk factor for the development of refractory AOSD, potentially reflecting higher disease activity or more extensive organ involvement in these patients.

Additionally, a review of prognosis and treatment options for AOSD patients suggests that early use of glucocorticoids, in combination with immunosuppressants and/or biologics, is crucial. However, there are several limitations to our study. First, it was a single-center study with a relatively small sample size, and we were unable to analyze cytokine profiles, which are important in AOSD, due to insufficient data. Second, we did not classify the prognosis of patients based on whether they had refractory disease, a distinction that will be addressed in future studies. Third, we did not examine the relationship between different treatments and outcomes in patients with refractory AOSD, but we plan to analyze these factors after collecting additional data.

In conclusion, our study is the first to demonstrate that PLT count, NLR, and the AOSD system score are independent predictors of risk factors in patients with refractory AOSD. Given the wide availability of these markers, our findings could easily be integrated into clinical practice, offering early warnings for clinicians to predict the progression of AOSD, which could ultimately improve patient outcomes.

## Figures and Tables

**Table 1:** Clinical characteristics of AOSD patients.

**Characteristics**	**n** = 105
Age, M (P25, P75) (years)	36.00 (27.00, 54.50)
Disease duration, M (P25, P75) (days)	15.00 (10.00, 21.00)
Gender (male), no. (%)	32 (30.48%)
Fever, no. (%)	105 (100.00%)
Arthralgia, no. (%)	78 (74.29%)
Rash, no. (%)	99 (94.29%)
Sore throat, no. (%)	80 (76.19%)
Myalgia, no. (%)	59 (56.19%)
Lymphadenopathy, no. (%)	80 (76.19%)
Splenomegaly, no. (%)	35 (33.33%)
Liver damage, no. (%)	27 (25.71%)
Lung involvement, no. (%)	43 (40.95%)
Heart involvement, no. (%)	18 (17.14%)
MAS, no. (%)	25 (23.81%)
Comorbidities, no. (%)	25 (23.81%)
WBCs, M (P25, P75) (× 10^9^/L)	13.06 (9.83, 16.95)
NEU, M (P25, P75) (× 10^9^/L)	10.90 (7.60, 14.72)
Lymphocyte count, M (P25, P75) (× 10^9^/L)	1.19 (0.78, 1.78)
NLR, M (P25, P75)	7.72 (5.09, 13.85)
PLT, M (P25, P75) (× 10^9^/L)	281.00 (194.00, 348.00)
PLR, M (P25, P75)	225.15 (150.19, 338.15)
ALT, M (P25, P75) (U/L)	39.00 (23.00, 80.50)
AST, M (P25, P75) (U/L)	42.00 (26.00, 66.00)
ALP, M (P25, P75) (U/L)	95.00 (80.00, 139.00)
*γ*-GT, M (P25, P75) (U/L)	42.00 (23.50, 104.00)
LDH, M (P25, P75) (U/L)	351.00 (275.00, 500.00)
CRP, M (P25, P75) (mg/L)	94.50 (51.25, 173.15)
ESR, M (P25, P75) (mm/h)	40.00 (18.00, 62.00)
SF, M (P25, P75) (ng/mL)	2865.00 (864.25, 7550.75)
FBG, M (P25, P75) (g/L)	5.33 (4.01, 6.45)
D-dimer, M (P25, P75) (*μ*g/mL)	3.62 (1.59, 5.97)
AOSD system score, M (P25, P75)	5.00 (5.00, 6.50)

Abbreviations: ALP, alkaline phosphatase; ALT, alanine aminotransferase; AST, aspartate aminotransferase; CRP, C-reactive protein; ESR, erythrocyte sedimentation rate; FBG, fibrinogen; LDH, lactic dehydrogenase; NEU, neutrophil count; NLR, neutrophil-to-lymphocyte ratio; PLR, platelet to lymphocyte ratio; PLT, platelet; SF, serum ferritin; WBCs, white blood cells; *γ*-GT, *γ*-glutamyl transferase.

**Table 2:** Clinical characteristics of refractory and nonrefractory AOSD patients.

**Variable**	**Refractory group (** **n** = 41**)**	**Non-refractory group (** **n** = 64**)**	**χ** ^2^/**Z**	**p** ** value**
Age, M (P25, P75) (years)	34.00 (25.50, 53.00)	37.50 (28.00, 54.75)	−0.920	0. 358
Gender (male), no. (%)	11 (26.83%)	21 (32.81%)	0.422	0.516
Disease duration, M (P25, P75) (days)	15.00 (9.50, 20.00)	15.00 (10.00, 27.75)	−0.699	0.484
Fever, no. (%)	41 (100.00%)	64 (100.00%)	—	—
Sore throat, no. (%)	34 (82.93%)	46 (71.88%)	1.683	0.195
Arthrodynia, no. (%)	27 (65.85%)	51 (79.69%)	2.504	0.114
Myalgia, no. (%)	21 (51.22%)	38 (59.38%)	0.675	0.411
Skin rash, no. (%)	40 (97.56%)	59 (92.19%)	0.528	0.468
Lymphadenopathy, no. (%)	34 (82.93%)	46 (71.88%)	1.683	0.195
Splenomegaly, no. (%)	20 (48.78%)	15 (23.44%)	7.223	0.007
Liver damage, no. (%)	13 (31.71%)	14 (21.88%)	1.265	0.261
Lung involvement, no. (%)	17 (41.46%)	26 (40.63%)	0.007	0.932
Heart involvement, no. (%)	8 (19.51%)	10 (15.63%)	0.266	0.606
MAS, no. (%)	25 (60.98%)	0 (0.00%)	—	0.000
Comorbidities, no. (%)	9 (21.95%)	16 (25.00%)	0.128	0.720
WBCs, M (P25, P75) (× 10^9^/L)	13.22 (10.06, 18.44)	12.98 (9.57, 16.37)	−0.522	0.602
NEU, M (P25, P75) (× 10^9^/L)	10.95 (7.99, 16.49)	10.85 (7.48, 13.53)	−0.778	0.436
Lymphocyte count, M (P25, P75) (× 10^9^/L)	1.05 (0.56, 1.40)	1.34 (1.00, 2.08)	−3.143	0.002
NLR	10.76 (6.95, 23.25)	7.20 (4.95, 10.54)	−2.933	0.003
PLT, M (P25, P75) (× 10^9^/L)	249 (172.50, 305.00)	301.50 (214.75, 390.00)	−2.733	0.006
PLR	272.73 (172.86, 379.04)	203.85 (141.18, 298.72)	−1.573	0.116
ALT, M (P25, P75) (U/L)	44.00 (27.00, 105.00)	36.00 (18.25, 76.00)	−1.557	0.119
AST, M (P25, P75) (U/L)	45.00 (29.50, 84.00)	40.00 (26.00, 61.00)	−1.682	0.093
ALP, M (P25, P75) (U/L)	99.00 (86.00, 170.50)	93.00 (76.50, 126.250)	−1.422	0.155
*γ*-GT, M (P25, P75) (U/L)	54.00 (24.50, 127.50)	42.00 (25.00, 99.50)	−0.677	0.498
LDH, M (P25, P75) (U/L)	402.50 (302.00, 585.00)	337.00 (253.75, 453.25)	−2.308	0.021
CRP, M (P25, P75) (mg/L)	115.70 (54.30, 202.26)	87.01 (48.27, 144.22)	−1.550	0.121
ESR, M (P25, P75) (mm/h)	47.00 (37.00, 64.50)	39.00 (22.00, 62.00)	−1.050	0.294
SF, M (P25, P75) (ng/mL)	6776.00 (2721.50, 11,975.50)	1831.00 (724.10, 3970.50)	−4.312	0.000
FBG, M (P25, P75) (g/L)	5.86 (4.01, 6.69)	5.15 (3.94, 6.35)	−1.217	0.224
D-dimer, M (P25, P75) (*μ*g/mL)	4.54 (2.00, 7.74)	3.52 (1.55, 5.09)	−1.172	0.241
AOSD system score, M (P25, P75)	5.50 (5.00, 7.00)	6.00 (5.50, 8.00)	−2.364	0.018

Abbreviations: ALP, alkaline phosphatase; ALT, alanine aminotransferase; AST, aspartate aminotransferase; CRP, C-reactive protein; ESR, erythrocyte sedimentation rate; FBG, fibrinogen; LDH, lactic dehydrogenase; NEU, neutrophil count; NLR, neutrophil-to-lymphocyte ratio; PLR, platelet-to-lymphocyte ratio; PLT, platelet; SF, serum ferritin; WBCs, white blood cells; *γ*-GT, *γ*-glutamyl transferase.

**Table 3:** Multivariate logistic regression predicts risk factors of refractory AOSD.

**Variables**	**B**	**S** **E**	**Wald**	**OR (95% CI)**	**p** ** value**
PLT (× 10^9^/L)	−0.007	0.002	9.154	0.993 (0.988, 0.997)	0.002
NLR	0.058	0.026	5.134	1.060 (1.008, 1.114)	0.023
AOSD system score	0.360	0.162	4.959	1.433 (1.044, 1.967)	0.026

Abbreviations: NLR, neutrophil-to-lymphocyte ratio; PLT, platelet.

**Table 4:** Diagnostic values of laboratory characteristics relevant to refractory AOSD.

**Variables**	**AUC**	**Optimum cutoff value**	**Youden index**	**95% CI**	**p** ** value**	**Sensitivity (%)**	**Specificity (%)**
PLT (× 10^9^/L)	0.659	314.5	0.274	(0.555, 0.762)	0.006	80.5	46.9
NLR	0.661	10.555	0.287	(0.571, 0.789)	0.006	53.7	75.0
AOSD system score	0.660	5.5	0.256	(0.555, 0.765)	0.006	75.6	50.0

Abbreviations: CI, confidence interval; NLR, neutrophil-to-lymphocyte ratio; PLT, platelet.

## Data Availability

The data that support the findings of this study are available from the corresponding author upon reasonable request.
